# Form and Function of the skin glands in the Himalayan newt *Tylototriton verrucosus*

**DOI:** 10.1186/s40851-018-0095-x

**Published:** 2018-06-13

**Authors:** Marion Wanninger, Thomas Schwaha, Egon Heiss

**Affiliations:** 1 Department of Integrative Zoology, University of Vienna, Althanstr, 14, A-1090 Vienna, Austria; 2 Institute of Zoology and Evolutionary Research, Friedrich-Schiller-University of Jena, Erbertstr. 1, 07743 Jena, Germany

**Keywords:** Salamander, Skin glands, Poison glands, Anti-predator adaptation

## Abstract

**Background:**

Amphibians have evolved a remarkable diversity of defensive mechanisms against predators. One of the most conspicuous components in their defense is related to their ability to produce and store a high variety of bioactive (noxious to poisonous) substances in specialized skin glands. Previous studies have shown that *T. verrucosus* is poisonous with the potential to truly harm or even kill would-be predators by the effect of its toxic skin secretions. However, little is known on form and function of the skin glands responsible for production and release of these secretions.

**Results:**

By using light- and scanning electron microscopy along with confocal laser scanning microscopy, we show that *T. verrucosus* exhibits three different multicellular skin glands: one mucous- and two granular glands. While mucous glands are responsible for the production of the slippery mucus, granular glands are considered the production site of toxins. The first type of granular glands (GG1) is found throughout the skin, though its average size can vary between body regions. The second type of granular glands (GG2) can reach larger dimensions compared with the former type and is restricted to the tail region. Despite their different morphology, all three skin gland types are enwrapped by a distinct myoepithelial sheath that is more prominently developed in the granular (i.e. poison-) glands compared to the mucous glands. The myoepithelial sheath consists of one layer of regularly arranged slender myoepithelial cells that run from the gland pore to the basal gland pole.

**Conclusions:**

This study shows that the skin in the Himalayan newt *T. verrucosus* displays one mucus- and two poison gland types enwrapped by a myoepithelial sheath. Contraction of the myoepithelium squeezes the glands and glandular content is released upon the skin surface where the secretion can deploy its defensive potential.

## Background

One of the main components of lissamphibian skin is the glandular tissue, the products of which are involved in a variety of functions [25, 29]. Two types of dermal glands – granular and mucous – are present in all adult extant lissamphibians studied to date (e.g. [20, 25, 28, 40, 47, 48, 57, 69]). Skin gland secretions are released onto the body surface (e.g. [22, 36, 42]) and can be used for a variety of protective purposes. Skin secretions can make the body surface slippery to facilitate escape from aggressors [67] or contain natural ‘super glues’, used to immobilize a predator [4, 27, 72]. Some components of lissamphibian skin secretions contain antimicrobial peptides as protection against a variety of microbial pathogens (e.g. [64, 74]). Other secretions are unpleasant tasting, irritating or even toxic, making the amphibian unpalatable to predators [8, 10, 18, 69]. Accordingly, amphibians produce a remarkable diversity of bioactive substances in their skin glands; more than 100 bioactive peptides, 30 bioactive amines, and over 800 alkaloids have been isolated from amphibian skin secretions [16, 18, 26, 30, 51, 65].

Mucous and granular glands can be distinguished by morphological and histochemical properties. Mucous glands are smaller than granular glands and widely distributed throughout the integument and secrete their contents in an apocrine to merocrine way continuously onto the skin surface [37]. The mucous secretion plays an important role, as it regulates water loss, acts as barrier against pathogens, is an important lubricant, reduces friction under water, and minimizes mechanical damage to the skin when out of the water [28].

Granular glands, the second type of lissamphibian skin glands are found throughout the skin, but some areas show higher concentrations of enlarged granular gland fields [2, 9, 11, 13, 25, 31, 41, 50, 51]. Although granular glands can differ substantially in form and content amongst lissamphibians, they are generally acinar in nature and are built up by giant cells containing granular material that often fuse into syncytia [19, 23, 24, 28, 29, 33, 34, 52, 62, 69]. Granular glands are capable of synthesizing and secreting mainly bioactive substances such as amines, peptides, and alkaloids [26] and are considered the site of skin toxin production and storage (e.g. [1, 3, 17, 18, 21, 55, 56, 58, 61, 68, 69]).

The granular glands can discharge their contents onto the body surface within seconds [52]. The secretory material is released from the gland through an apical pore in a holocrine manner (e.g. [22, 39, 52, 54, 63]). In most lissamphibians, the granular gland secretions are, if not poisonous, at least harsh and irritating to mucous membranes and useful in deterring potential predators (e.g. [10, 11, 25, 36]). The granular gland secretions of most salamanders are noxious to toxic, and are able to truly harm or even kill a would-be predator [9, 10, 12, 32, 46, 70]. Accordingly, noxious skin secretions have been considered to be the most important tools for repelling predators in salamanders, and most other antipredator adaptations, such as antipredator posturing or aposematic coloration, are dependent upon release and storage sites of skin secretions. Within salamandrids, *Tylototriton verrucosus*, has been reported to be highly poisonous; its skin secretions show a LD_50_ value (tested intraperitoneally on mice) comparable with that of some viper toxins (for overview see [11, 46]). However, little is known on the structure of its skin glands and their functional role in defense.

Accordingly, this study aims at providing new details on form and function of the cutaneous glands in *T. verrucosus* by using light, scanning electron and confocal laser scanning microscopy and by discussing the morphological results in a functional context. We show that (i) there are three different gland types that can be structurally distinguished: one mucous and two granular glands, as previously described in the closely related newt *Pleurodeles waltl* [34], (ii) all three gland types are enwrapped by a distinct myoepithelial sheath and (iii) there are distinct body regions with accumulations of enlarged granular glands that are actively displayed during defense.

## Methods

Five adult individuals (three females, two males) *Tylototriton verrucosus* were examined for the present study. Total body-length ranged from 115 to 130 mm and body weight ranged from 12.4 g to 18.2 g. The newts were obtained commercially and kept in a 250-l tank with 12 h /12 h light:dark cycle. Animals were fed twice a week with earthworms, bloodworms and fish pieces. For morphological investigations the newts were anesthetized with a 0.05% aqueous solution of MS222 (protocol after [15]), decapitated and immersed into fixation solution. Skin samples from the head (parotoids), dorsal trunk, lateral trunk (lateral wart region), ventral trunk as well as the dorsal and ventral part of the tail were removed for further micro-anatomical analyses described below.

### Scanning electron microscopy (SEM)

For scanning electron microscopy, samples were fixed in Bouin’s-solution [7] for 4 weeks, rinsed in 70% ethanol and dehydrated in a graded ethanol series. Next, samples were transferred into acetone and dried in a critical point drying machine (Polaron, Watford, UK). The dried samples were then coated with gold in an AGAR B7340 sputtercoater (Agar Scientific Ltd., Stansted, UK) and observed using a Philips XL-20 scanning electron microscope (Philips, Eindhoven, NL).

### Light microscopy (LM)

Light microscopy analyses included paraffin based histological investigations as well as investigations of semi thin sections. In addition to standard paraffin-based histology, resin embedding and semi thin sectioning was necessary to keep structural integrity of samples with high amount of glandular tissue. For paraffin-based histology, samples were fixed in Bouin’s solution for 4 weeks after which we followed standard protocols described elsewhere (e.g. [7, 45]). Histological sections were mounted on glass slides and stained with Haematoxylin-Eosin (HE), Heidenhain’s AZAN trichrome stain, Alcian blue (AB) at pH 2.5, periodic acid Schiff (PAS) and Coomassie Brilliant Blue (CBB) (all standard staining procedures after [7, 45]). Observations and photographic documentations were performed with a Nikon Eclipse 800 (Nikon, Tokyo, Japan) and Axiolab (Carl Zeiss Jena, Germany) compound microscope.

For semi-thin sectioning, two fixation and embedding methods were used. For the first procedure, samples were fixed in Bouin’s solution [7], rinsed in 70% ethanol, dehydrated in a graded ethanol series and embedded in LR white embedding resin (Ted Pella, Inc., Redding, California, USA) that was polymerized at 60 °C for 20 h. For the second procedure, samples were fixed in modified Karnovsky-solution [44] (2.5% glutaraldehyde and 2% formaldehyde in 0.1 M cacodylate buffer). After rinsing in 0.1 M cacodylate buffer, samples were postfixed for 2 h in buffered 1% osmium tetroxide at room temperature. This was followed by dehydration in a graded ethanol and acetone series, embedding in Agar 100 Resin (Agar Scientific, Essex, UK) and polymerization at 65 °C for 15 h. The embedded and polymerized samples from both methods were then cut into 1 μm thin sections on a Reichert Ultracut S microtome (Leica, Wetzlar, Germany) using diamond histo-knives (Diatome AG, Biel, Switzerland). The semi-thin sections were mounted on glass slides, stained with toluidine blue and documented as described above for histological sections.

### Confocal laser scanning microscopy (CLSM)

For muscular stainings, skin samples from the parotoid, trunk and tail area were fixed in 4% paraformaldehyde in 0.1 M phosphate buffer (PB) for 1 h at room temperature. Samples were afterwards rinsed three times in PB and stored in PB containing 0.1% NaN3 at 4 °C until further procedure. Prior to staining samples were embedded in gelatine-ovalbumine solution before they were sectioned on a Leica VT1200S vibratome (Leica Microsystems, Wetzlar, Germany) at a thickness of 150–200 μm. For staining, samples were incubated in a 1:40 solution of AlexaFluor-488 phalloidin in 4% Triton-X solution in PB. A drop of DAPI (4′, 6-diamidino-2-phenylindole) was added for counterstaining of the cell nuclei. Staining lasted for approximately 1 day at room temperature. Samples were afterwards rinsed three times in PB for 30 min each before they were mounted in Fluoromount G (Southern Biotech, Birmingham, Alabama, USA). Confocal image stacks were taken with a Leica SP5II confocal laser scanning microscope (Leica Microsystems, Wetzlar, Germany). Image stacks were afterwards processed with Amira 6.0 (FEI, Hillsboro, USA).

### Histomorphometry and statistics

We tested for granular gland size differences (i) between the two gland types GG1 (granular gland type 1) and GG2 (granular gland type 2) and (ii) between six body regions: parotoids, ventral trunk, lateral trunk (wart region), dorsal trunk, dorsal tail and ventral tail. To this end, we measured maximum height and width in a total of 300 glands (50 GG from each region listed above) by using the measurement-tool-kit of the vector-based open source software Inkscape (https://inkscape.org/de/). To account for the irregular tri-axial ellipsoid shape of the glands from which only two dimensions are known (i.e. height and width), we used the average of height and width as the most appropriate measure to compare the gland sizes. Because the data violated the assumptions for parametric tests (i.e. homogeneity and normal distribution of the variables’ residuals also after log10 transformation), we performed the non-parametric Mann-Whitney-U-Test to test for size differences between gland types (GG1 vs. GG2) and the Kruskal-Wallis approach to test for glandular size differences between regions (parotoids, dorsal trunk, lateral trunk, ventral trunk, dorsal tail, ventral tail).

In a second approach, we tested for granule (vesicle) size differences between GG1 and GG2. We measured the diameter in 100 randomly selected granules in each gland type. Given that the granules were regularly roundish, the diameter was considered the most appropriate measure to describe their size. As the data violated the assumptions for parametric statistics, we performed the non-parametric Mann-Whitney-U-Test to test for size differences of granules across gland types.

All statistical analyses were performed with Microsoft Excel 2010 and SPSS Statistics 20 software packages.

Animal keeping and all procedures involving living animals were in strict accordance with the Austrian Protection of Animals Act and the study was approved by the University of Vienna Advisory Board of Study Affairs.

## Results

### Scanning electron microscopy

**Fig. 1:**
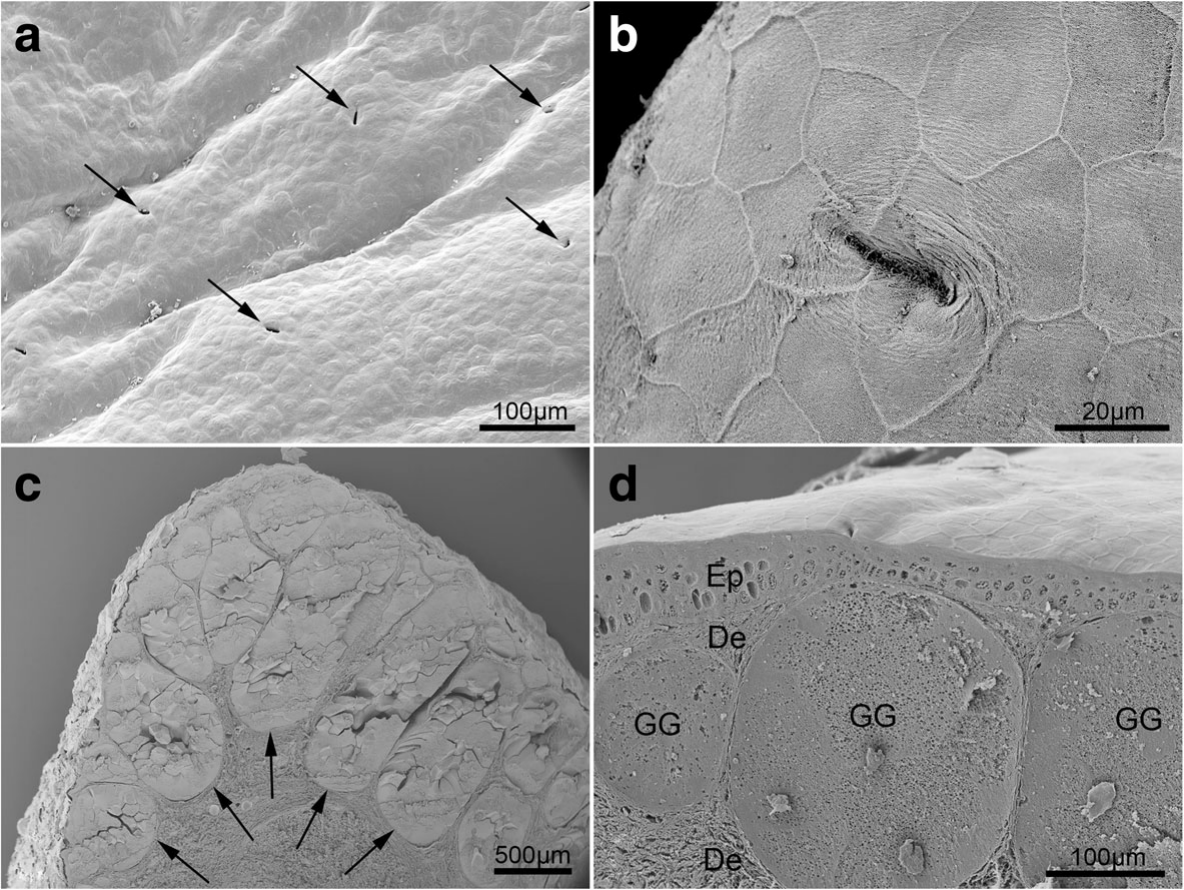
Scanning electron micrographs showing the skin surface (**a**, **b**) and sections through the skin (**c**, **d**) of *T. verrucosus*. **a** Overview of the skin surface in the dorsal trunk region with numerous gland pores (indicated by arrows). **b** Detail showing a single gland pore and surrounding epithelial cells. **c** Section through the left parotoid gland. Note the densely packed granular (poison) glands indicated by arrows. **d** Section through the dorsal trunk region with roundish poison glands just beneath the epithelium. De, dermis; Ep, epithelium, GG, granular gland

Scanning electron microscopic investigations included the examination of the skin surface, as well as observations of skin cross-sections. The skin surface in *T. verrucosus* was relatively flat but bore numerous pores of cutaneous glands (Fig. 1a, b). Higher magnification revealed the slender, slit-like gland pores surrounded by flattened, irregularly hexagonal-shaped keratinocytes (Fig. 1b). Sections through the skin revealed the two main skin layers, namely the epithelium and the dermis. While the epithelium was thin and was built up by few cell layers, the dermis was thick, mainly consisted of fibrous material and housed the balloon-like shaped main bodies of the skin glands (Fig. 1d). The main bodies of the glands embedded in the spongy dermis were connected to the exterior through a short glandular duct that traversed the epithelium and ended up in the gland-pore on the skin surface. SEM allowed fast examination of many skin-regions and it became apparent that high densities of enlarged cutaneous glands were present in some regions, while others bore discrete numbers of moderately sized glands (Fig. 1c, d). The most impressive concentrations were found in the parotoid region (Fig. 1c) and the dorsal tail.

### Light microscopy

The skin of *T. verrucosus* showed a large number of cutaneous glands, which were scattered throughout the dermis (Fig. 1a-e). All cutaneous gland types shared some common features as they were multicellular and simple acinar in shape. In general, one mucous and two distinct granular gland types could be identified in *T. verrucosus* as they were characterized by distinct structural and histochemical properties.

**Fig. 2:**
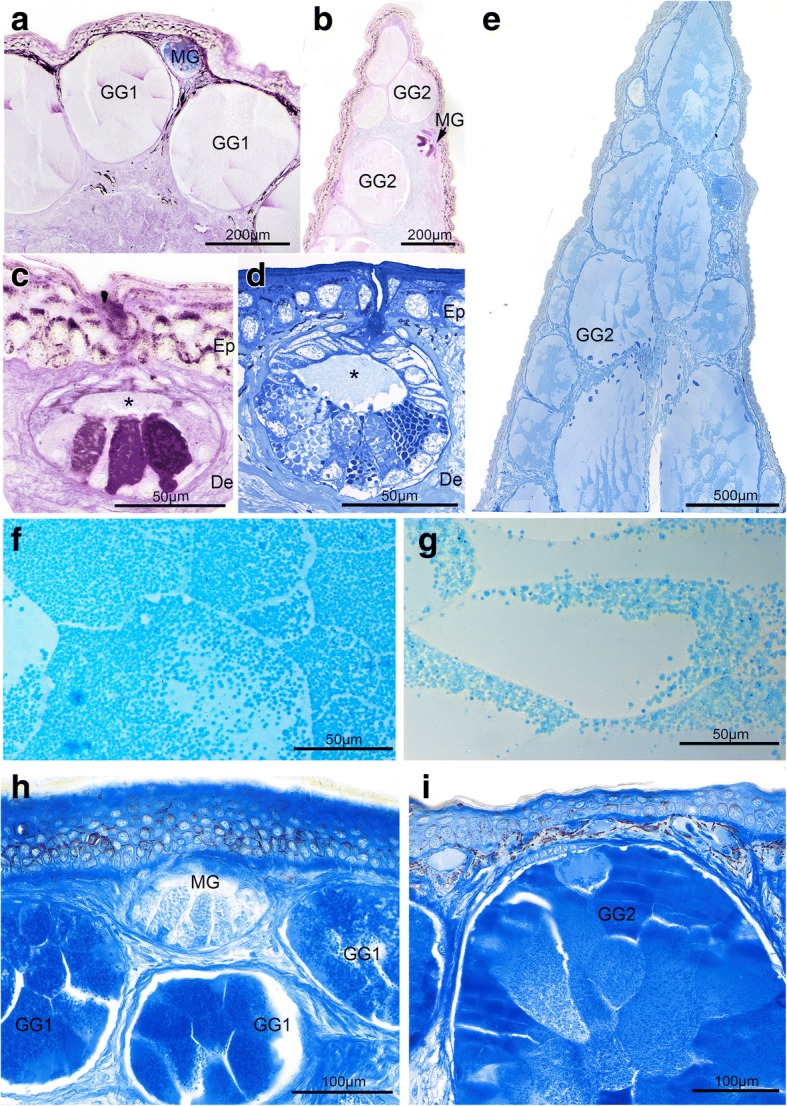
Light micrographs of acinar dermal glands in the skin of *T. verrucosus*. **a**-**c**. Sections stained with Alcian blue at pH 2.5 and Periodic acid Schiff (AB-PAS) showing **a** the back region with large granular glands type 1 (GG1) extending deep into the dermis and a conspicuously smaller mucous gland (MG), **b** the dorsal tail edge with large granular glands type 2 (GG2) next to a MG and **c** details of a MG. Note that GG1s and GG2s neither react to the AB nor to the PAS test, while the MGs react both to AB and PAS (MG-lumen in C indicated by an asterisk). **d-g**. Toluidine blue stainings showing: **d**. Details of a MG with a central lumen (indicated by asterisk) surrounded by smaller cells in the apical half and large cells filled with secretory material in the basal half of the gland. **e**. Cross section through the dorsal tail edge, ca. 3 cm distal from the tail base showing the densely arranged giant granular glands type 2 (GG2). **e** and **f**. Details of GG1 (**e**) and GG2 (**f**) illustrating the differently arranged granules in the two gland types. H and I: Coomassie Brilliant Blue (CBB) stained sections from the lateral trunk (**h**) and ventral tail (**i**) showing positive CBB staining of GG1 (**h**) and GG2 (**i**) and negative CBB-reaction of MG (**h**). Ep, epithelium; De, dermis

Mucous glands (MG) were found all over the skin as small simple acinar glands with a wide lumen that was continuous with the secretory duct of the gland (Fig. 2a, c, d). The lumen was encircled by secretory cells. The secretory cells in the apical gland pole, close to the pore, were smallest, but increased in size towards the lateral walls and were largest in the basal part of the gland (Fig. 2c, d). Basally located secretory cells ranged from tall and pyramidal to cuboidal in shape with a proximally located round nucleus. The cytoplasm of the basally located MG-cells was entirely filled with large flocculent to granular secretory products. Within a given gland, the histochemical properties of the secretory content often varied considerably between cells (Fig. 2a–c, h). In general, most of the cells within the mucous gland stained strongly PAS positive – pink to purple (Fig. 2b, c) – but some cells within the mucous glands also reacted positive to the AB-test at pH 2.5 (Fig. 2a), while others neither reacted to the PAS nor to the AB-test (Fig. 2c). All mucous glands reacted negatively to the CBB-test for proteins (Fig. 2h). Accordingly, mucous glands in *T. verrucosus* mainly produce and store neutral to slightly acidic mucopolysaccharid components.

The second cutaneous gland type in *T. verrucosus* was represented by the granular glands. They were roundish to oval in shape, large in size and could extend through the whole spongy dermis. Two types of granular glands could be distinguished based on structural differences. The first type, termed here granular gland type 1 (GG1), was found in all skin samples examined (but were very rare in the tail), whereas the second type was restricted to the dorsal and ventral tail edges. This second type is named granular gland type 2 (GG2) and will be treated separately.

GG1s were present throughout the skin (Fig. 2a, h). In general, these glands appeared roundish but apparently shape depended on the available space in the stratum spongiosum of the dermis. In the parotoid region, where GG1s were densely packed, they were rather oval and, in relation to the skin surface oriented perpendicularly. The neck region represented the intercalated tract between secretory unit and duct and had a thick wall of undifferentiated cells. The duct itself was lined by keratinocytes and opened to the exterior. GG1 consisted of densely packed, large secretory cells that filled the entire gland with remarkable amounts of dense granules (Fig. 2f). These glands were composed of single secretory cells of homogenous appearance, with flat nuclei positioned in the gland periphery. The secretory cells were filled with similarly sized and similarly shaped granules. No cellular fusions into syncytia were observed. In general, the GG1 were eosinophilic, reacted negatively to the Alcian blue test at pH 2.5 and the PAS-test (Fig. 2a), but positive to the CBB-test (Fig. 2h). Though ordinary sized GG1 could be found throughout the skin in *T. verrucosus*, their size differed between body regions. While the largest GG1 were found in the parotoid region and the dorsal trunk, they were smallest on the ventral trunk (see also statistics results).

The GG2 glands were only found in samples taken from the tail. They were large, deeply embedded in the spongy dermal layer and irregular elliptical in shape (Fig. 2b, e). In some sections, especially from the dorsal tail edge, the GG2s were so abundant and large that they represented the most prominent tissue of the region (Fig. 2e). The main body of GG2 was composed of densely packed secretory cells that were separated by clearly visible cell membranes and no syncytial organization was observed (Fig. 2f). In general, GG2s were eosinophilic, reacted negatively for the AB test at pH 2.5 and the PAS test (Fig. 2b). The positive Coomassie Brilliant Blue staining further pointed to the presence of proteinaceous secretory material (Fig. 2i). The secretory granules were all similar in shape and size, but unlike those in GG1, they were not distributed homogenously throughout the cells, but restricted to distinct areas that formed granular patches (Fig. 2f, g).

### Confocal laser scanning microscopy (CLSM)

**Fig. 3:**
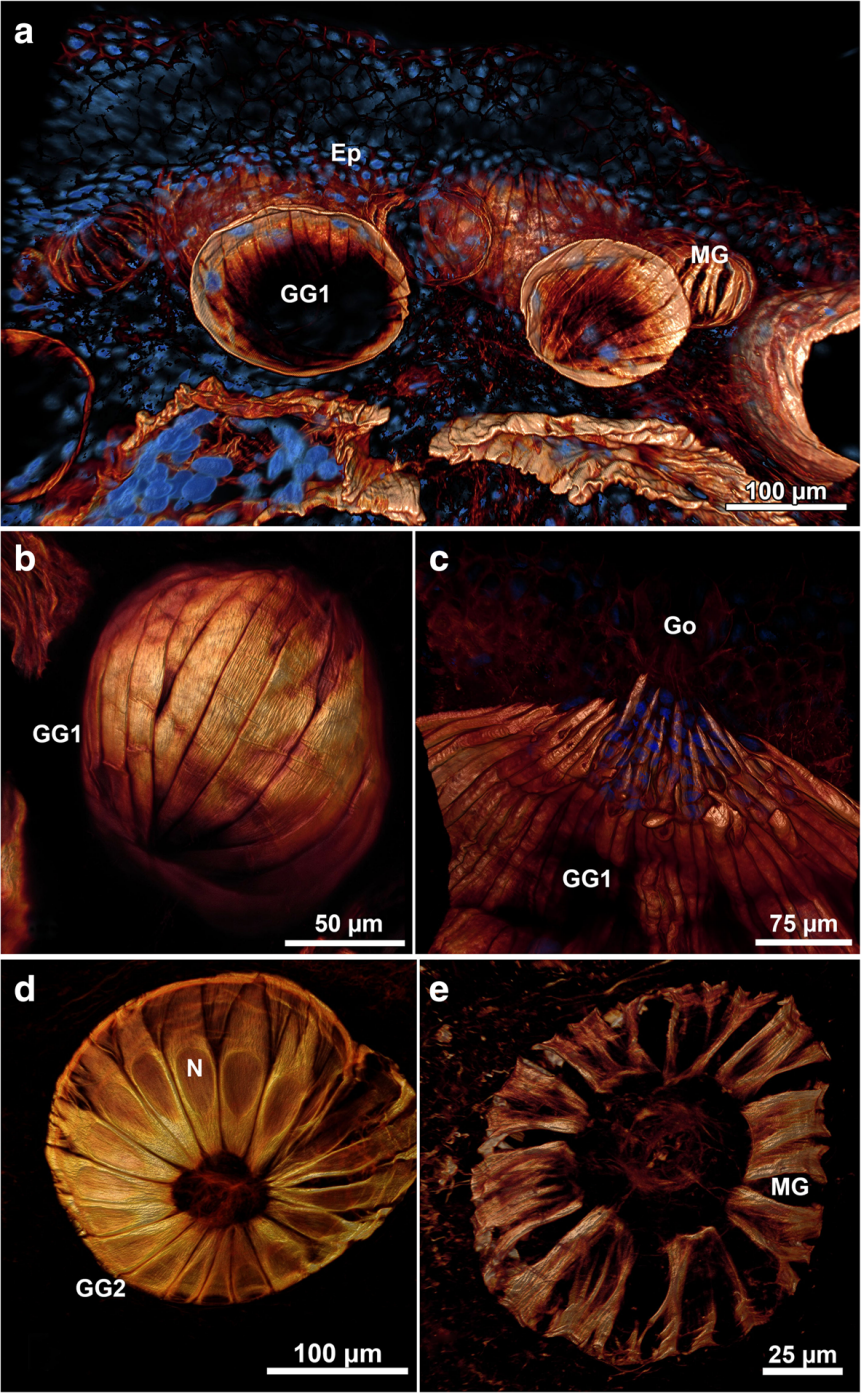
Muscular system of the cutaneous glands of *Tylototriton verrucosus*. F-actin staining by AlexaFluor-conjugated phalloidin on vibratome sections and confocal laser scanning microscopy. **a** Overview of the trunk region showing the epidermis on top as well as the smaller mucus glands and the large granular glands type 1 (GG1). **b** Detail of a GG1 in the parotoid area. The musculature forms a regular basket around the gland with the fibres orientated in parallel perpendicular to the surface of the skin. **c** Detail of the opening of a GG1. The myoepithelial cells commonly extend as thin fibres towards the gland opening. **d** Top view of a granular gland type 2 (GG2) of the tail region showing a similar arrangement of cells as in the first type of granular gland. Note the oval areas of the nuclei regularly arranged close to the gland opening. **e** Detail of the muscular system of a mucus gland in the tail region, top view similar as in (**d**). The muscular basket of the mucus glands commonly looks less dense than the granular types. Ep, epidermis; Go, gland opening; MG, mucus gland; N, nucleus

CLSM revealed the 3D arrangement of the myoepithelium that enwrapped all three gland types (Fig. 3). Phalloidin labeling of actin filaments revealed the regularly arranged slender and flattened myoepithelial cells running from the basal gland pole to the glandular duct region where their distal ends are arranged around the glandular opening. The myoepithelium was present in all three multicellular gland types and showed a regular arrangement to form a continuous and closed myoepithelial sheath in GG1 and GG2. In contrast, it was a comparatively irregular and discontinuous cover in mucous glands (Fig. 3a, d).

### Statistics

(i) Size differences between granular gland types and regions

The Mann-Whitney-U-Test revealed significant differences between the two gland types GG1 and GG2 (U = − 4.2; *P* < 0.001) where GG2 with an averaged diameter of 472 ± 162 μm (mean ± sd) were larger than GG1 (396 ± 146 μm). The Kruskal-Wallis-Test revealed significant differences between regions (H = 100.2; *P* < 0.001) and descriptive statists showed that the largest granular glands were those in the dorsal tail region with an averaged diameter of 559 ± 172 μm, followed by GG in the parotoid region (473 ± 165 μm), the dorsal trunk (420 ± 137 μm), the lateral trunk (417 ± 121 μm), the ventral tail (385 ± 87 μm) and the ventral trunk (276 ± 121 μm).

(ii) Granule size differences between GG types

The Mann-Whitney-U-Test revealed significant differences of granule size between GG1 and GG2 (U = − 8.5; *P* < 0.001) where granules in GG1 with a diameter of 1.5 ± 0.27 μm were smaller than granules in GG2 (2.1 ± 0.5 μm).

## Discussion

Our study shows that the entire skin in *T. verrucosus* is interspersed with granular (GG) and mucous glands (MG). GGs share some common features with MGs, as they are multicellular acinar glandular structures located in the dermis and open to the skin surface by an excretory duct. Both glands are built up by the neck region, the secretory unit, and the surrounding myoepithelium. On the other hand, GGs are significantly larger than MGs and lack a distinct lumen. Furthermore, GGs mainly contain protein components and no significant levels of mucopolysaccharides, while MGs mainly contain mucopolysaccharides but no significant levels of protein components. However, some cells within MGs neither reacted positively to AB-PAS nor to the CBB-test, implying that they neither contain mucopolysaccharides nor protein components. Given that MGs continuously proliferate their secretory cells, which undergo a maturation process [14], the lack of positive reaction may point to an immature state of the unstained MG cells.

In contrast to MGs, GGs in *T. verrucosus* mainly produce protein materials and Brodie Jr et al., [11] and Lai et al. [46] showed the high toxicity of the GG proteins in toxicological experiments on mice. In contrast, von Byern et al. [73] demonstrated the high biocompatibility of *T. verrucosus* skin secretions in a series of cell culture experiments. Von Byern et al. [73] argued that the biocompatibility in the in vitro experiments might be related to the presence of the peptide tylotoin previously isolated from *Tylototriton* skin [53]. In fact, tylotoin was shown to enhance cell proliferation [53, 73], which could positively affect cell survival and cell grow in the in vitro studies. Accordingly, the GG-proteins of *T. verrucosus* seem to have no negative effect on cell cultures, but are highly toxic when tested on more complex animal models. However, detailed biochemical analyses are needed to shed light on the GG components and their function.

Amongst the GGs in *T. verrucosus*, two distinct types can be distinguished based on morphological features (GG1 and GG2). GG1s are on average smaller and can be found throughout the body, while the larger GG2s are only present in restricted areas of the skin: the dorsal and ventral edges of the tail. Despite morphological differences, both GGs in *T. verrucosus* show similar histochemical properties (AB-negative, PAS-negative, CBB-positive) pointing towards similar chemical components. Given similar components in both glands, the active use of the tail studded with GG2s in defensive behavior [11] and the fact that GG1 are definitely assigned as poison glands (e.g. [1, 3, 17, 55, 69, 71]), we suggest that GG2 represent a second poison-gland type in *T. verrucosus*, the function of which is related to defense.

Although GG1 and GG2 on average differ in size, glandular size is also largely dependent on the body region in which they occur. Some body regions are characterized by the presence of fields of enlarged GGs and similar as in *T. verrucosus*, conspicuous regions of enlarged granular gland accumulations were also reported in the tail, the parotoid area and on the lateral and dorsal trunk in other salamanders where they appear as ‘glandular warts’ [9–11, 25]. Such clusters of GGs are closely related to specific defense behaviors. Specifically, it has been shown that when threatened, many salamanders tilt glandular regions toward the stimulus (e.g. [10]). Such postures may increase the chance for the predator to first contact the most unpalatable parts of the salamander’s body, making the initial encounter maximally unpleasant for the predator, which learns to avoid similar prey [10, 58]. In fact, behavioral experiments of Brodie Jr et al. [11] have shown that when a threatening stimulus was applied, *T. verrucosus* released skin secretion and adopted a variety of body postures where the head was depressed, the body arched, the ribs rotated anteriorly (‘erected’) and the tail elevated and coiled above the body or undulated and slashed towards the threatening stimulus [11]. By depressing its head, *T. verrucosus* exposes the large parotoids with their large and densely packed GG1s. Similarly, when arching the body, *T. verrucosus* displays the concentration of the large GG1s of the dorsal trunk. Elevating, coiling and undulating the tail above the body further displays a region of concentrated large GG2s. Undulating the tail can attract the predator’s attention and if the predator then approaches the posterior body region of the salamander, *T. verrucosus* slashes its tail with its large poison glands into the aggressor [11].

For an efficient protective strategy, it is essential that glandular secretions are promptly available upon the skin surface and here, the contractile myoepithelium plays a major role. *Tylototriton verrucosus* has a well-developed myoepithelial sheath around MGs and GGs. While the myoepithelial sheath around the MGs is discontinuous and possibly of minor functional importance, the myoepithelial system with its densely packed smooth muscle cells around the GGs is key to enabling the rapid and active discharge of the glandular secretions. But how does the myoepithelium contribute to glandular discharge, and how is its function controlled? Sjöberg and Flock [66] showed that lissamphibian glandular myoepithelial cells are innervated by the sympathetic nervous system. Alarm or injury signals activate the sympathetic nervous system and neurotransmitters engage the alpha-adrenergic receptors, causing myoepithelial contraction (e.g. [5, 38, 60]). Myoepithelial contraction squeezes the acinar gland so that its contents are released onto the skin surface through the gland pore. Myoepithelial contraction can be inhibited by alpha receptor antagonists and experimental studies have shown that myoepithelial cell contraction is not necessarily an all-or-none reaction but instead is dependent on the concentration and type of stimulator and/or inhibitor released [38]. Accordingly, the total amount of gland secretion released onto the skin surface can be fine-tuned to meet the demands of a given stimulus or threat.

The active secretion of noxious substances upon the skin surface and the display of body regions of increased secretion to a potential predator have proven to be highly efficient defensive strategies. Some lissamphibians go further and have evolved mechanisms to deliver their toxic secretions into the blood stream of the aggressor [6, 10, 11, 35, 43, 58]. The African frogs *Astylosternus* and *Trichobatrachus* for example, have sectorial terminal phalanges on their hind limbs that can cut through the skin, which are used as bony claws to scratch any potential predator when attacked [6]. In theory, such scratches might allow toxic skin secretions to enter the aggressor’s blood stream by the wound, increasing the repellent effect of the frog skin secretions exponentially. Similarly, the Brazilian hylid frogs *Corythomantis greeningi* and *Aparasphenodon brunoi* have bony spines in their snout region that penetrate the skin in areas with concentrated granular glands and are used to injure the aggressor to deliver the toxins into its wound [43]. A convergent strategy is used by some salamandrid salamanders. The sister-taxon of *Tylototriton*, *Echinotriton* along with the more distantly related genus *Pleurodeles,* protrude their sharply pointed and elongated ribs through their skin to use them as stinging tools to cause small injuries to an attacking predator and pave the way for skin toxins to seep into the predators wound where they cause severe and potentially deathly intoxications [10, 11, 35, 49, 58]. Similarly to *Echinotriton* and *Pleurodeles*, *Tylototriton* has elongated ribs that are erected upon a threatening stimulus but the rib tips are blunt and do not penetrate the skin [59]. The blunt rib tips are located just below the lateral glandular warts and erection of the elongated ribs in *T. verrucosus* pushes against the dense arrangement of GG1 within the warts, what in turn supports action of the myoepithelium and helps to discharge the GG1s [11]. So though *T. verrucosus* has lost the ability to use its ribs as stinging tools, rib erection is still used in defense but the defensive strategy has shifted from a mechanical-chemical (such as in *Echinotriton* and *Pleurodeles*) to a merely chemical approach.

## Conclusions

This study provides new details on the morphology and distribution of multicellular skin glands, and quantifies their regional size differences in *Tylototriton verrucosus*, a salamandrid newt known for its toxic skin secretions. We show three different multicellular skin gland types and argue that two of them are poison glands that increase in size and density in body regions actively displayed during the stereotypic defensive behavior. All skin glands studied here are enwrapped by a distinct myoepithelial sheath whose contraction enables rapid expulsion of the glandular content for active use against potential predators.
